# Pore Structure Characterization of Sodium Hydroxide Activated Slag Using Mercury Intrusion Porosimetry, Nitrogen Adsorption, and Image Analysis

**DOI:** 10.3390/ma11061035

**Published:** 2018-06-19

**Authors:** Yibing Zuo, Guang Ye

**Affiliations:** Microlab, Faculty of Civil Engineering and Geosciences, Delft University of Technology, Stevinweg 1, 2628 CN Delft, The Netherlands; G.Ye@tudelft.nl

**Keywords:** NaOH, slag, pore structure, MIP, N_2_ adsorption, image analysis

## Abstract

The pore structure of alkali-activated slag has a significant influence on its performance. However, the literature shows insufficient studies regarding the suitability of different techniques for characterizing the pore structure and the influences of Na_2_O and curing age on pore structure development. In pursuit of a better understanding, the pore structure of sodium hydroxide activated slag paste was characterized by multiple techniques, e.g., mercury intrusion porosimetry (MIP), nitrogen (N_2_) adsorption, and scanning electron microscopy (SEM) image analysis. The sodium hydroxide activated slag pastes were prepared with three different contents of Na_2_O (Na_2_O/slag = 4, 6, and 8%) and cured for different times up to 360 days. The microstructure observation reveals that outer C–(N–)A–S–H and inner C–(N–)A–S–H grow successively around the reacting slag grains, along with crystalline reaction products which are formed in the empty coarse pore space. The increase of Na_2_O content and curing age lead to a finer pore structure. The MIP measurements show that the total porosity drops about 70% within the first day, and that one peak at most, corresponding to gel pores, was identified in the differential curves of all the investigated samples from 1 to 360 days. On the contrary, only one peak, corresponding to capillary pores, was identified by SEM-image analysis. The differential curves derived from N_2_ adsorption generally reveal two peaks, and the trend that the pore diameters of those two peaks vary with curing age depends on the content of Na_2_O. Compared to Portland cement, sodium hydroxide activated slag has a higher pore space filling capacity (*χ*, *V_products_*/*V_slag-reacted_*), while the capacity decreases with increasing Na_2_O content and curing age.

## 1. Introduction

When ground granulated blast furnace slag is brought into contact with an alkaline activator, the slag grains start to dissolve and a set of reactions commence, resulting in various solid reaction products. In alkali-activated slag, the reaction products are usually categorized into primary reaction products and secondary reaction products [1]. The primary reaction product is a type of calcium-sodium aluminosilicate hydrates (C–(N–)A–S–H) [2,3]. The secondary reaction products are crystalline phases, such as hydrotalcite [3], tetracalcium aluminate hydrate [4], katoite [5], and stratlingite [6], etc. The empty or initially liquid-filled space is called capillary pore. With continuous reaction of slag, capillary pores are gradually filled by the reaction products. As such, capillary pores are gradually blocked and the sizes are refined with time. The sizes of capillary pores are in the range from several nanometers (nm) to several micrometers (μm). Similar to calcium silicate hydrates (C–S–H) in Portland cement-based materials, C–(N–)A–S–H in alkali-activated slag is a type of gels with low crystallinity that contain pores [7,8]. The pores in C–(N–)A–S–H are called gel pores. The gel pore has a size of approximately several nanometers. Therefore, the alkali-activated slag, as any other cement-based material, is a porous material that contains two different pore systems—capillary pores and gel pores.

The pore structure of alkali-activated slag has a significant influence on its performance. In general, the parameters that are usually used to characterize pore structure are total porosity (volume fraction of pores relative to the bulk volume of sample), pore size distribution, and pore connectivity. Like other cement-based materials, many properties of alkali-activated slag are directly or indirectly related to those pore structure parameters [9,10,11]. The total porosity has a substantial effect on strength, fracture energy, toughness, and elasticity. The pore size distribution influences shrinkage and creep properties. The total porosity along with pore connectivity determines water and ionic transport and thus governs the permeability, diffusivity, and durability. Therefore, pore structure characterization is of great significance to interpret the properties and to evaluate the service life of alkali-activated slag concrete.

For the characterization of the pore structure of cement-based materials, several techniques have been used in the past decades, such as mercury intrusion porosimetry (MIP) [12], X-ray computed microtomography [13], nitrogen (N_2_) adsorption [14], proton nuclear magnetic resonance relaxometry [15], and scanning electron microscopy (SEM) image analysis [16]. Among those techniques, MIP, N_2_ adsorption, and image analysis are the most commonly used [17]. MIP is able to determine nearly the whole range of pore sizes (from several nanometers to hundreds of micrometers). Nitrogen adsorption technique enables nanoscale analysis of pores. Image analysis allows the characterization of pores from a real SEM image of the material. Among those three techniques, each one has its advantages and limitations [17,18,19,20]. In order to characterize the full image of pore structure, it is usually needed to use the three techniques simultaneously.

By using MIP to study the pore structure, Collins and Sanjayan found that alkali-activated slag paste had a much higher proportion of pore sizes within the mesopore region (1.25 to 25 nm) than comparable ordinary Portland cement paste [11]. Palacios and Puertas used MIP to study the pore structure of alkali-activated slag mortars with and without shrinkage-reducing admixtures [21]. Those researchers found that the admixtures modified the pore structure by increasing the percentage of pores with diameters ranging from 1.0 to 0.1 μm. In another study on the alkali-activated slag mortars, the pore structure observations obtained from MIP measurements were in agreement with strength results and water permeability results [10]. Ismail et al. used the N_2_ adsorption method to study the pore structure of alkali-activated slag pastes prepared with different drying methods and found that solvent treatment was the best way to preserve the microstructure of samples [22]. By using the SEM-image analysis method, Ben Haha et al. found that the main reduction in coarse porosity (0.1 to 5 μm) of sodium hydroxide activated slag paste took place within the first day, decreasing from about 55% to about 19% [23].

The literature shows two aspects that are not sufficiently studied on the pore structure of alkali-activated slag. Firstly, since combinations of MIP, N_2_ adsorption, and image analysis are rarely employed to study the pore structure, the suitability of each technique in characterizing the pores in alkali-activated slag is unknown. Secondly, there are few studies carried out to investigate the long-term performance of alkali-activated slag in terms of microstructure formation and pore structure development with curing age up to 360 days. As a result, the influences of Na_2_O content and curing age on the pore structure development are not well understood.

In pursuit of a better understanding, this study combined the techniques of MIP, N_2_ adsorption, and SEM-image analysis to investigate the pore structure of sodium hydroxide activated slag paste. The sodium hydroxide activated slag pastes were prepared with three different contents of Na_2_O (Na_2_O/slag = 4, 6, and 8%) and cured for different times up to 360 days. MIP and N_2_ adsorption were used to determine the pore structure of samples at 1, 7, 28, 180, and 360 days, while SEM image analysis was used to detect the pore structure of samples at 1, 7, and 28 days. The pore structure data derived from those three different techniques were presented, discussed, and critically compared. The influences of Na_2_O content and curing age on the pore structure were investigated in terms of total porosity, pore size distribution, differential curves of pore size distribution, ink-bottle porosity, pore connectivity, and pore space filling capacity. Based on the experimental results and discussion, conceptual models were used to describe the microstructure formation process of sodium hydroxide activated slag.

## 2. Materials and Methods

### 2.1. Materials and Mixtures

**Figure 1:**
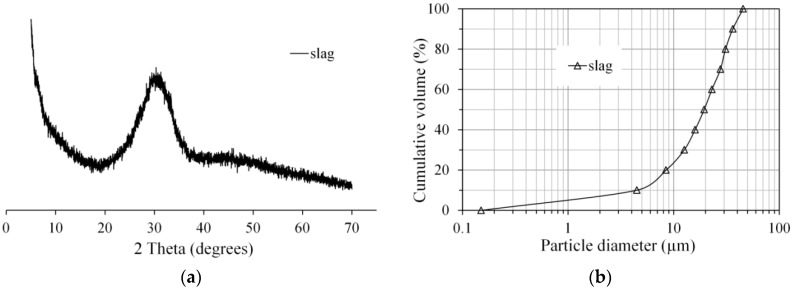
The X-ray diffraction pattern (**a**) and particle size distribution (**b**) of slag, determined by a powder diffractometer and laser diffraction respectively.

Ground granulated blast furnace slag was used in this study to prepare sodium hydroxide activated slag paste. The chemical composition of slag was determined by X-ray fluorescence spectrometry (XRF), as listed in Table 1. Figure 1 presents the X-ray diffraction pattern and particle size distribution of slag, determined by a powder diffractometer and laser diffraction respectively. Sodium hydroxide (analytical grade, >98%) was mixed with distilled water to prepare the sodium hydroxide activator. The mix compositions of the pastes are listed in Table 2. The mixtures were denoted as AAS4, AAS6, and AAS8, corresponding to the mixtures with Na_2_O/slag = 4, 6, and 8%, respectively.

The slag and sodium hydroxide activator were mixed in a commercial Hobart mixer with two minutes of low-speed mixing, followed by two minutes of high-speed mixing. Subsequently, the mixed slag paste was cast into commercial cylinder polyethylene jars (d = 35 mm and h = 70 mm) and vibrated for 30 s on a vibrating table. All the samples were stored in closed jars at room temperature until the designed testing age. At the designed testing age, the samples were crushed into small pieces with dimensions of 1–2 cm^3^, and then the small pieces of samples were immersed in isopropanol for at least two weeks to stop reaction of slag. After that, the samples were dried in a vacuum at 20 °C for one week. Then, the vacuum dried samples were stored in a desiccator until testing.

### 2.2. Methods

#### 2.2.1. MIP

**Figure 2:**
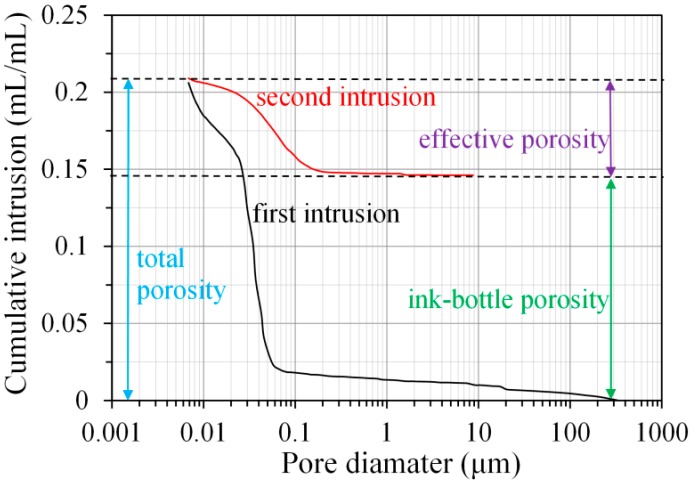
Typical cumulative pore size distribution curves obtained from the first and second intrusion process in mercury intrusion porosimetry (MIP) measurement.

In this study, MIP measurements were carried out with a Micrometrics PoreSizer^®^ 9500 (micromeritics, Brussel, Belgium) with a maximum intrusion pressure of 210 MPa. In each measurement, the test was conducted in two stages. The first stage is the low-pressure stage with the pressure running from 0 to 0.14 MPa. The second stage is the high-pressure stage. The intrusion procedure starts with the pressure running from 0.14 to 210 MPa. After the applied pressure reaching the highest pressure (210 MPa), i.e., the end of intrusion procedure, the extrusion procedure starts with the applied pressure decreasing to 0.17 MPa. In the high-pressure stage, two cycles of intrusion and extrusion procedures were conducted in the MIP measurements in this study. By assuming the pores are cylindrical, the diameter of pores intruded by mercury at each pressure step was calculated using the Washburn equation [24] as follows:

(1) $$D=\frac{-4\gamma \cos\theta }{P}$$

where *D* is the pore diameter of material, *γ* is the surface tension of mercury (0.485 N/m used here [25]), *θ* is the contact angle between mercury and sample surface, and *P* is the applied pressure. In this study, the contact angle was taken as 138° for the calculation of pore size distribution during mercury intrusion process [26]. Figure 2 shows the typical cumulative pore size distribution (PSD) curves obtained from the first and second intrusion curves in MIP test. In the figure, the total porosity, effective porosity, and ink-bottle porosity are schematically illustrated.

• Total porosity

The total volume of pores is defined as the total volume of mercury intruded at the maximum applied pressure in the first intrusion. Then, the total porosity is calculated as the total volume of pores divided by the bulk volume of the sample.

• Effective porosity

The effective porosity is calculated from the volume of mercury intruded at the maximum applied pressure in the second intrusion divided by the bulk volume of the sample.

• Ink-bottle porosity

After extrusion, the mercury in the continuous pores is extruded from the sample; however, the mercury in the “ink-bottle” pores [12] will remain inside. The ink-bottle porosity is calculated by subtracting the effective porosity from the total porosity.

• Pore connectivity

Pore connectivity is a key parameter that influences the transport properties of cementitious materials. The pore connectivity (*η*) can be calculated as the quotient of the effective porosity (*ϕ_e_*) over the total porosity (*ϕ_t_*) [27,28], as follows:

(2) $$\eta =\frac{\phi_{e}}{\phi_{t}}\times 100\%$$

#### 2.2.2. N_2_ Adsorption

N_2_ adsorption is able to detect small pores (from 0.3 to 300 nm [29]) in porous materials. With this technique, the pores of the alkali-activated slag paste in the nanoscale can be analyzed. In this study, the nitrogen adsorption tests were performed by using Gemini VII 2390 (micromeritics, Brussel, Belgium) with a relative pressure range from 0.05 to 0.998. The relative pressure is defined as the equilibrium vapor pressure divided by the saturation vapor pressure. Based on the nitrogen adsorption data, Barrett–Joyner–Halenda (BJH) model [30] was used to derive the pore size distribution curve. 

#### 2.2.3. SEM-Image Analysis

The SEM-image analysis was performed based on the images obtained from environmental scanning electron microscope (ESEM). The vacuum dried samples were impregnated using a low viscosity epoxy resin and then polished down to ¼ μm [31]. Then, the polished samples were examined by ESEM (Philips XL30, Eindhoven, The Netherland) with backscattering electron (BSE) mode at an acceleration voltage of 20 kV under low vacuum mode. The water vapor pressure was kept at 1.0 Torr. The physical size of each image was 254 μm in length and 190 μm in width. The magnification of each image was 500×, and the image size was 1424 × 968 pixel. By calculation, the resolution of the image was 0.1786 μm/pixel.

**Figure 3:**
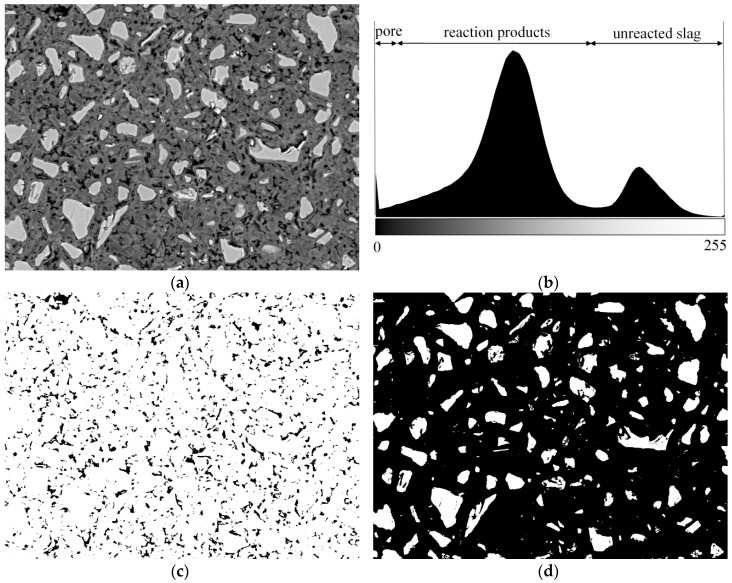
A typical backscattering electron (BSE) image (**a**) with its grey level histogram (**b**), pores (**c**), and unreacted slag (**d**) obtained after applying thresholds on the original BSE image.

A typical BSE image is shown in Figure 3a and its grey level histogram is shown in Figure 3b. According to the grey level histogram, a threshold was applied on the original BSE image and the pores (distinguished as black) were obtained as shown in Figure 3c. The pores obtained in such a way will be submitted to the calculation of pore size distribution of samples. Similarly, by applying a threshold on the original BSE image, the unreacted slag (distinguished as white) was obtained as shown in Figure 3d, and the area fraction of unreacted slag can be obtained. In the image analysis, it is usually assumed that the area fractions calculated from a two-dimensional image are equal to the volume fractions in three dimensions [17,32]. As such, the degree of reaction of slag can be calculated as follows:

(3) $$\alpha \left(t\right)=\left(1-\frac{F_{t}}{F_{0}}\right)\times 100\%$$

where *α*(*t*) is the degree of reaction of slag at age *t*, *F_t_* is the volume fraction of unreacted slag at age *t* (obtained from image analysis), and *F*_0_ is the initial volume fraction of slag. According to the mixture design, the initial volume fraction of slag was calculated as 45.8% for AAS4, AAS6, and AAS8.

**Figure 4:**
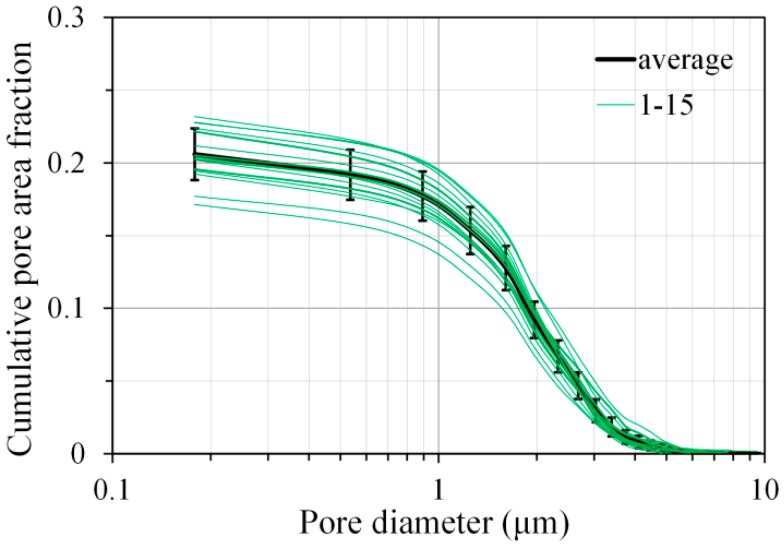
Calculated pore size distribution of samples for AAS4 at curing age of 1 day (AAS indicates alkali-activated slag, the number following AAS refers to the weight percentage of Na_2_O with respect to slag. The water to slag ratio was 0.4 and the curing temperature was 20 °C).

Yang et al. developed an image-based algorithm to obtain the pore size distribution of porous media [33]. This algorithm was employed in this study to calculate the pore size distribution based on the pores obtained from the BSE image. It is reported that the image analysis based on 12 or more BSE images could enable a 95% degree of confidence [17]. In this study, a total number of 15 images on randomly selected locations for each sample were submitted to image analysis, and the averaged pore size distribution was used as the final pore size distribution for the sample, as shown in Figure 4 for AAS4 at 1 day.

#### 2.2.4. Solid Phase Growth

The volume fraction (*F_solid_*) of solid phase in sodium hydroxide activated slag sample can be expressed as follows:

(4) $$F_{solid}=\frac{V_{slag-unreacted}+V_{products}}{V_{sample}}$$

where *V_slag-unreacted_*, *V_products_*, and *V_sample_* are the volumes of unreacted slag, products, and sample, respectively. It is noted that the volume of gel pores in C–(N–)A–S–H is not included in *V_products_*. *V_slag-unreacted_*, and *V_products_* can be given as follows:

(5) $$V_{slag-unreacted}=V_{slag}-V_{slag-reacted}$$

(6) $$V_{products}=\chi \cdot V_{slag-reacted}$$

where *V_slag_* and *V_slag-reacted_* are the volumes of slag at the beginning and reacted slag, *χ* is the volume ratio of products relative to the reacted slag.

By substituting Equations (5) and (6) into Equation (4), *F_solid_* can be obtained as:

(7) $$F_{solid}=\frac{V_{slag}}{V_{sample}}\cdot \left(1+\left(\chi -1\right)\frac{V_{slag-reacted}}{V_{slag}}\right)$$

Compared to the bulk volume of sample, the volume change due to autogenous shrinkage can be neglected. Then, the volume of the sample can be regarded as constant and thus equal to the initial volume of sample. So, Equation (7) can be rewritten as:

(8) $$F_{solid}=\left(1-P_{0}\right)\cdot \left(1+\left(\chi -1\right)\cdot \alpha \right)$$

where *P*_0_ is the initial porosity and *α* is the degree of reaction of slag. According to Equation (8), the total porosity (*P*) can be expressed as:

(9) $$P=1-\left(1-P_{0}\right)\cdot \left(1+\left(\chi -1\right)\cdot \alpha \right)$$

As such, the volume ratio of the products relative to the reacted slag can be determined as:

(10) $$\chi =1+\frac{1-P}{\alpha \cdot \left(1-P_{0}\right)}-\frac{1}{\alpha }$$

In this study, *χ* was used as an indicator to study the solid phase growth in sodium hydroxide activated slag samples. This indicator represents the pore space filling capacity by solid phase.

## 3. Results

### 3.1. Morphology of Hardened Sodium Hydroxide Activated Slag Samples

**Figure 5:**
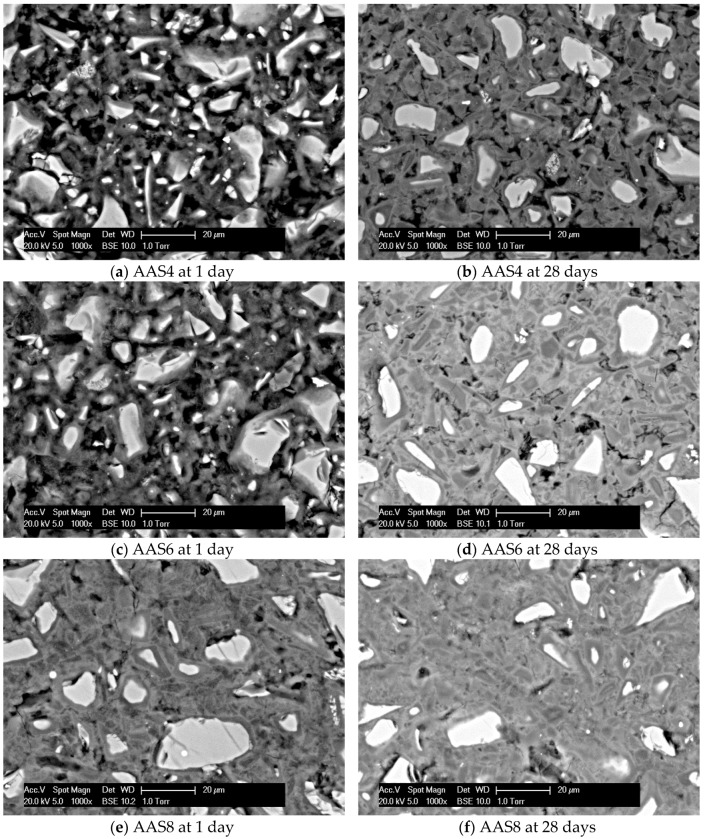
Microstructure of samples for AAS4 (**a**,**b**), AAS6 (**c**,**d**), and AAS8 (**e**,**f**) at 1 and 28 days. In the graphs, AAS indicates alkali-activated slag, the number following AAS refers to the weight percentage of Na_2_O with respect to slag. For all samples, the water to slag ratio was 0.4 and the curing temperature was 20 °C.

Figure 5 shows the SEM-micrographs of the sodium hydroxide activated slag samples at 1 and 28 days. It is clear that for all the samples, grey reaction products surround the bright unreacted slag grains. As the curing age and Na_2_O content increase, the sample exhibits a larger volume of reaction products and hence a denser microstructure. It is noted that two-tone layers of reaction products are found surrounding the unreacted slag grains for the sample with longer curing time and higher Na_2_O content, such as AAS4 at 28 days (see Figure 4b) and AAS8 at 1 day (see Figure 4e). This observation is also reported in another study [23]. The layer formed later, e.g., inner products, is darker than the layer formed earlier, e.g., outer products. The difference in grey level is due to the different nanoporosities and, therefore, to different densities and backscattered electron coefficients [23]. This point will be further discussed in Section 4.4.

### 3.2. Degree of Reaction of Slag

**Figure 6:**
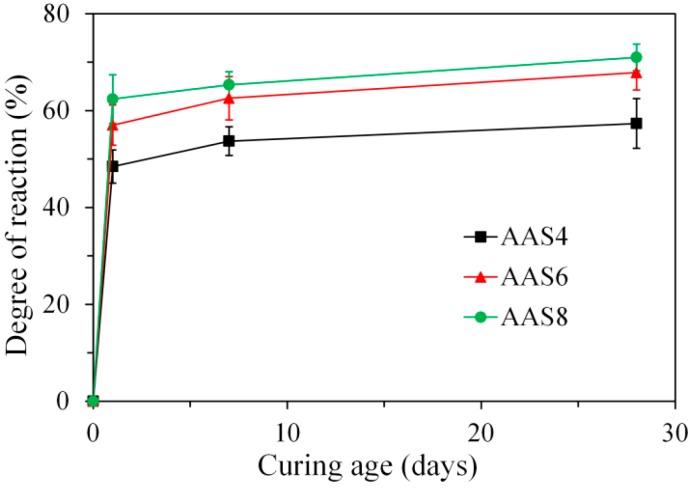
Degree of reaction of slag, derived from image analysis, for AAS4, AAS6, and AAS8. In the graphs, AAS indicates alkali-activated slag, and the number following AAS refers to the weight percentage of Na_2_O with respect to slag. For all samples, the water to slag ratio was 0.4 and the curing temperature was 20 °C.

By image analysis (see Equation (3)), the degree of reaction of slag was derived as shown in Figure 6. It is clear that the increase of curing age and Na_2_O content led to a higher degree of reaction. Particularly, the degree of reaction reaches 48.5%, 57.0%, and 62.4% at 1 day for AAS4, AAS6, and AAS8 respectively. After 1 day, the degree of reaction increases slowly with time.

### 3.3. Pore Structure of Sodium Hydroxide Activated Slag Pastes Determined by MIP

#### 3.3.1. Total Porosity Derived from MIP

**Figure 7:**
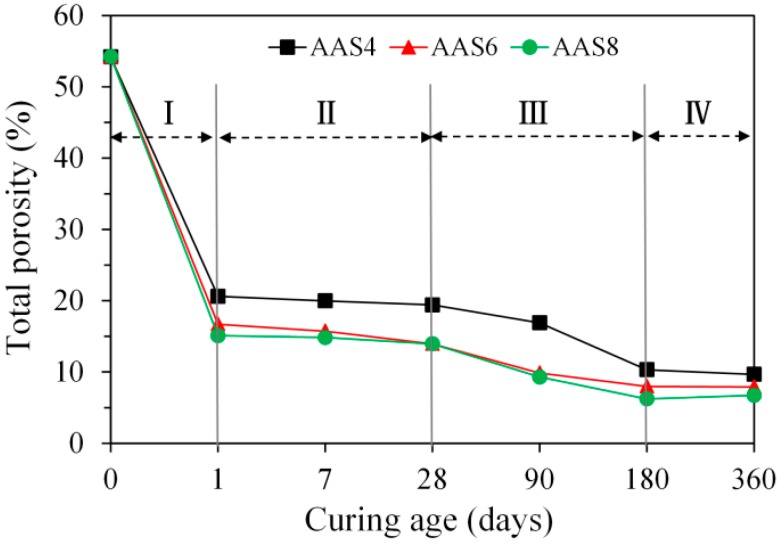
The total porosity of samples as a function of time up to 360 days, derived from MIP. In the graphs, I, II, III, and IV represent four stages, AAS indicates alkali-activated slag, and the number following AAS refers to the weight percentage of Na_2_O with respect to slag. For all samples, the water to slag ratio was 0.4 and the curing temperature was 20 °C.

Figure 7 presents the total porosity of samples as a function of time up to 360 days for AAS4, AAS6, and AAS8, derived from MIP. According to the mixture design, the total porosity of samples at the initial moment when slag contacts the alkaline activator was calculated as 54.2%. As the reaction of slag proceeds, the total porosity of samples decreases continuously with time. According to the development of total porosity with time, four stages can be identified, as seen in Figure 7. In stage I, from the beginning when slag contacts the alkaline activator, the total porosity drops considerably (about 70%). The main reduction in total porosity takes place within the first day, which is consistent with the findings in Ref. [23]. In stage II from 1 to 28 days, the total porosity decreases very slightly. During the whole period in the second stage, the reduction of total porosity is within an interval of 5.1%. In stage III from 28 to 180 days, the total porosity decreases a little bit faster when compared to the previous stage. In the last stage (IV) from 180 to 360 days, the total porosity decreases very little with time. After 360 days of curing, the total porosity of the samples were 9.7%, 7.9%, and 6.7% for AAS4, AAS6, and AAS8 respectively. As the Na_2_O content increases from 4% (AAS4) to 6% (AAS6), the total porosity decreases about 28% after 1 day. However, a further increase of Na_2_O content from 6% (AAS6) to 8% (AAS8) does not lead to a significant decrease of the total porosity.

#### 3.3.2. Pore Size Distribution Derived from MIP

**Figure 8:**
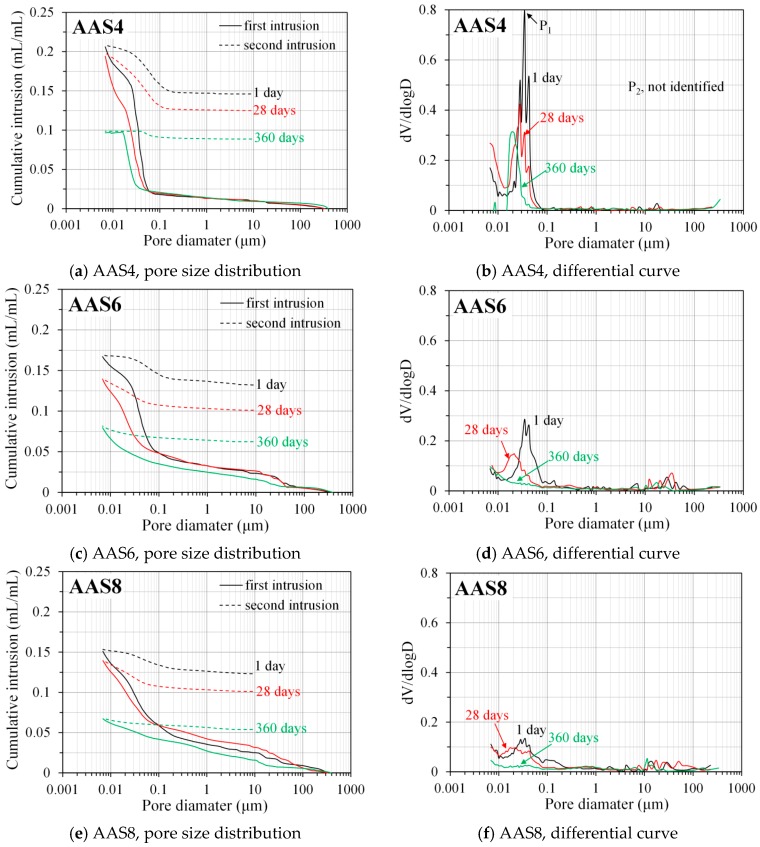
Pore size distribution and differential curves, derived from MIP, for AAS4 (**a**,**b**), AAS6 (**c**,**d**), and AAS8 (**e**,**f**) at 1, 28, and 360 days (the data at 7, 90, and 180 days are shown in Figure S1, as included in the Supplementary Materials). In the graphs, P_1_ and P_2_ are the first and second peak respectively (here P_2_ is not identified), AAS indicates alkali-activated slag, the number following AAS refers to the weight percentage of Na_2_O with respect to slag. For all samples, the water to slag ratio was 0.4 and the curing temperature was 20 °C.

Figure 8 shows the pore size distribution and differential curves, derived from MIP, for AAS4, AAS6, and AAS8 at the curing ages of 1, 28, and 360 days. The MIP data at the curing ages of 7, 90, and 180 days are presented in the Supplementary Materials (see Figure S1). It is clear that the total porosity decreases and the pore size distribution curve shifts to smaller pore sizes as the curing age increases, indicating a denser microstructure of samples. The reduced porosity and refined microstructure is attributed to a higher reaction degree of samples at later curing age (see Figure 6). A higher reaction degree leads to more reaction products, filling the pore space and consequently resulting in a denser microstructure. As the Na_2_O content increases, for example from 4% (AAS4) to 6% (AAS6), reduced porosity and refined microstructure can also be found. This also results from a higher reaction degree of samples caused by higher Na_2_O content (see Figure 6).

In Portland cement paste, two peaks exist in the differential curve of MIP pore size distribution, corresponding to two different pore systems [12,34,35]. The first peak has a pore diameter between 0.01 and 0.1 μm, corresponding to the threshold pore diameter of the gel pore system, and the second peak, with a pore size larger than 0.1 μm, corresponds to the threshold pore diameter of the capillary pore system [17,18,36]. Here, the physical meaning of a threshold pore diameter is that the pores whose diameter is larger than this diameter can form no connected path through the sample [37].

In this study, however, only the first peak (P_1_) was identified in the differential curves of MIP pore size distribution of AAS4, AAS6, and AAS8 at all curing ages, as shown in Figure 8 and Figure S1 (right columns). The first peak, i.e., corresponding to gel pores, is found from 0.01 to 0.04 μm for AAS4, AAS6, and AAS8. As curing age increases, the first peak shifts to a smaller pore diameter and its intensity decreases. It is noted that the first peak disappears after 28 days for AAS6 and AAS8. As the Na_2_O content increases, for example from 4% (AAS4) to 6% (AAS6), similar observations can be found for the first peak, in that it shifts slightly to a smaller pore diameter and its intensity decreases.

**Figure 9:**
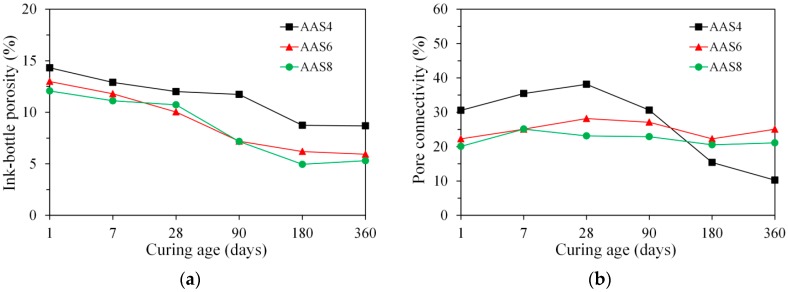
The ink-bottle porosity (**a**) and pore connectivity (**b**) as a function of time up to 360 days, derived from MIP. In the graphs, AAS indicates alkali-activated slag, and the number following AAS refers to the weight percentage of Na_2_O with respect to slag. For all samples, the water to slag ratio was 0.4 and the curing temperature was 20 °C.

The second peak (P_2_ > 0.1 μm) that corresponds to the threshold pore diameter of capillary pores is not identified in the differential curves. The absence of the second peak in the differential curves of MIP pore size distribution was also found for the cement paste (water/cement ratio = 0.4) after 180 days of curing, and for the blended cement pastes (30% fly ash, water/binder = 0.5; 50% fly ash, water/binder = 0.4) after 28 days of curing [37]. This suggests that sodium hydroxide activated slag samples at 1 day already have a very dense microstructure that is comparable to those of cement paste after 180 days of curing and the blended cement pastes after 28 days of curing. The absence of the second peak is mainly due to the fact that reaction products continuously grow into the pore space, resulting in a denser microstructure with the decrease of capillary porosity, smaller, and only partially connected capillary pores (pore connectivity < 1, as seen in Figure 9b). Consequently, the second peak becomes weak and even disappears when curing age increases. Besides, the “ink-bottle” effect may also lead to the absence of the second peak. This point will be further discussed in Section 4.1.

#### 3.3.3. Ink-Bottle Pore and Pore Connectivity Derived from MIP

The ink-bottle porosity is plotted as a function of time, as presented in Figure 9a. After 1 day, the ink-bottle porosity of samples is 14.3%, 13.0%, and 12.1% for AAS4, AAS6, and AAS8 respectively. With increasing curing age, the pore space of samples is gradually filled with reaction products. This results in the reduction of the volume of pores, including the ink-bottle pores. As such, the ink-bottle porosity of samples decreases continuously with time. An increase of Na_2_O content from 4% (AAS4) to 6% (AAS6) results in a smaller ink-bottle porosity, while a further increase from 6% (AAS6) to 8% (AAS8) shows no significant influence on the ink-bottle porosity.

Pore connectivity reflects the proportion of effective porosity in the total porosity. Figure 9b shows the plots of pore connectivity as a function of time up to 360 days for AAS4, AAS6, and AAS8. The pore connectivity increases with time up to 28 days of curing, after which it decreases continuously with time for AAS4 and AAS6. For AAS8, the pore connectivity increases from 1 day to 7 days and then decreases gradually with time until 360 days. The increase of pore connectivity at early age up to 28 days will be further discussed in Section 4.3. After 360 days of curing, the pore connectivity of samples is 10.2%, 25.1%, and 21.1% for AAS4, AAS6, and AAS8 respectively. Although AAS4 has a higher total porosity than AAS6 and AAS8, its pore connectivity is half of the pore connectivity of AAS6 and AAS8 at 360 days. This is because AAS4 has a larger ink-bottle porosity than AAS6 and AAS8.

### 3.4. Pore Structure of Sodium Hydroxide Activated Slag Pastes Determined by N_2_ Adsorption

**Figure 10:**
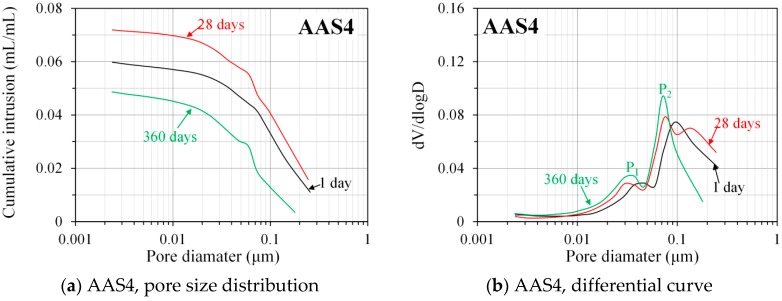

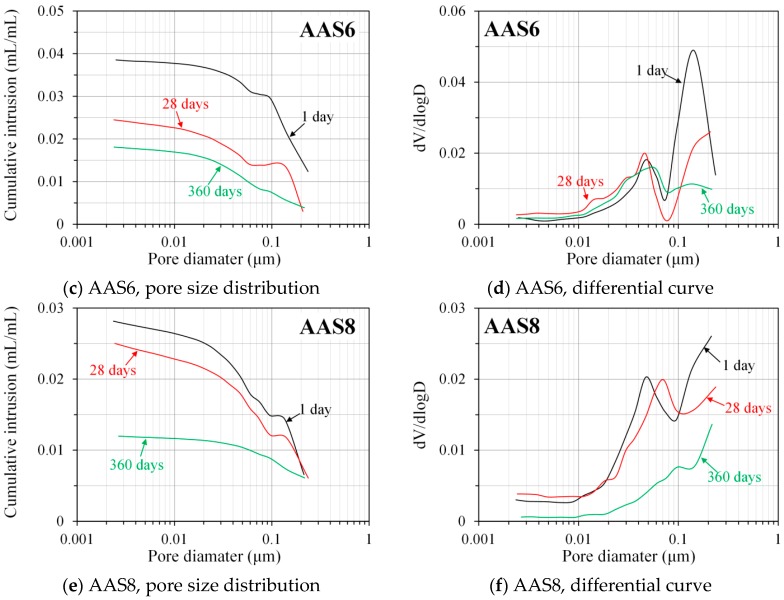
Pore size distribution and differential curves, derived from nitrogen adsorption, for AAS4 (**a**,**b**), AAS6 (**c**,**d**), and AAS8 (**e**,**f**) at 1, 28, and 360 days (the nitrogen adsorption data at 7 and 180 days are shown in Figure S2, as included in the Supplementary Materials). In the graphs, P_1_ and P_2_ are the first and second peak, respectively. AAS indicates alkali-activated slag, the number following AAS refers to the weight percentage of Na_2_O with respect to slag. For all samples, the water to slag ratio was 0.4 and the curing temperature was 20 °C.

Figure 10 shows the pore size distribution and differential curves, derived from nitrogen adsorption, for AAS4, AAS6, and AAS8 at the curing ages of 1, 28, and 360 days. The N_2_ adsorption data at the curing ages of 7 and 180 days are presented in the Supplementary Materials (see Figure S2). The pores detected by N_2_ adsorption are of sizes between 0.002 and 0.25 μm, which provides more detailed information on small pores (<0.25 μm) in the samples of alkali-activated slag. It can be observed that the porosity of small pores increases with time at the early age and then it decreases with time at a later age (also see Table 3). For example, the porosity of small pores in the sample of AAS4 increases at the early age up to 28 days, and then it decreases from 28 to 360 days. This result is expected and in agreement with the finding in Ref. [37]. At the early age, large pores are gradually filled by reaction products, resulting in the formation of small pores and consequently leading to an increase of the porosity of small pores. At a later age, large pores are hardly noticeable and reaction products are mainly formed in small pores. As such, the porosity of small pores starts to decrease with time. By comparing the porosity of small pores in the samples between AAS4, AAS6, and AAS8 (as listed in Table 3), it can be seen that an increase of Na_2_O content leads to a decrease of the porosity of small pores, showing a finer pore structure. This finding is in line with the results derived from MIP data.

In general, differential curves of the pore size distribution derived from N_2_ absorption show two peaks. It can be seen that the pore diameters corresponding to those two peaks vary with the curing age and content of Na_2_O. For the samples of AAS4, those two peaks are in the pore sizes between 0.01 and 0.1 μm. According to the definition of threshold pore diameter in Section 3.3.2, both of those two peaks correspond to gel pores. The first peak (P_1_) appears at a smaller pore diameter than the second peak (P_2_). As curing age increases, both peaks shift to smaller pore diameters and their intensities increase. For the AAS6 samples, the first peak appears at a pore diameter between 0.01 and 0.1 μm, while the second peak is in a pore size larger than 0.1 μm. Those two peaks correspond to gel pores and capillary pores, respectively. It seems that those two peaks show little shift as curing age increases. For the AAS8 samples, the first peak is readily seen at a pore diameter between 0.01 and 0.1 μm, corresponding to gel pores. On the contrary, the second peak is not readily seen but it is expected from development of the curve. The second peak appears at a pore diameter larger than 0.1 μm, corresponding to capillary pores. As curing age increases, the first peak shifts to a larger pore diameter.

### 3.5. Pore Structure of Sodium Hydroxide Activated Slag Pastes Characterized by Image Analysis

**Figure 11:**
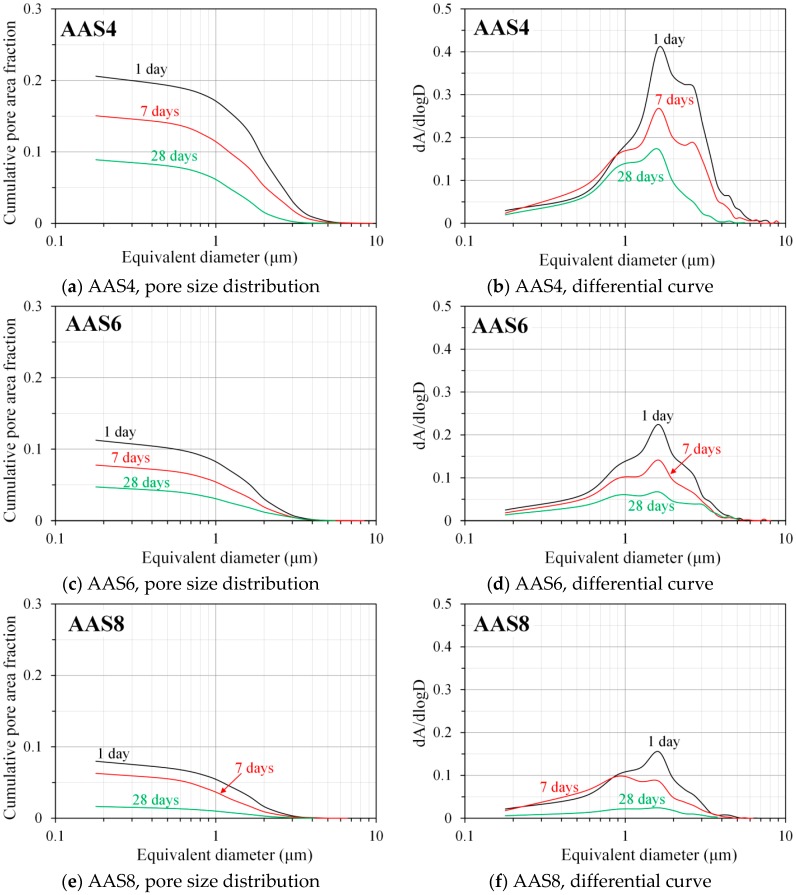
Pore size distribution and differential curves, derived from image analysis, for AAS4 (**a**,**b**), AAS6 (**c**,**d**), and AAS8 (**e**,**f**) at 1, 7, and 28 days. In the graphs, AAS indicates alkali-activated slag, the number following AAS refers to the weight percentage of Na_2_O with respect to slag. For all samples, the water to slag ratio was 0.4 and the curing temperature was 20 °C.

It should be borne in mind that the pore structure derived from image analysis only reveals the geometry of pores, but not the interconnectivity of the pore paths in the sample. Figure 11 shows the pore size distribution and differential curves derived from image analysis for AAS4, AAS6, and AAS8 at the curing ages of 1, 7, and 28 days. Similar to the total porosity determined by MIP, increases of curing age and Na_2_O content result in a lower total pore area. It indicates that the pores are finer for the sample with longer curing time and higher Na_2_O content, which is consistent with the observations based on the MIP results.

As seen in Figure 11 (right column), only the peak that corresponds to capillary pores is identified in the differential curves, which is different from the observation according to the differential curves derived from MIP. It is noted that the pore diameter that corresponds to the peak in Figure 11 (right column) does not have the physical meaning of a threshold pore diameter. The peak that corresponds to gel pores is not identified. This is because gel pores with average diameter smaller than 0.01 μm are about one order of magnitude smaller than the resolution of BSE imaging (0.1786 μm/pixel). As such, the image analysis based on BSE image cannot detect the gel pores. Similar to the first peak in the differential curve of MIP pore size distribution, increases of curing age and Na_2_O content also lead to a lower intensity of the peak in the differential curve derived from image analysis. However, the pore diameter corresponding to the peak derived from image analysis is found at about 1.6 μm, showing no change with increasing curing age and Na_2_O content. This is different from the observations by MIP that the threshold pore diameter of gel pores shifts to a smaller pore size as the curing age and Na_2_O content increase.

## 4. Discussion

### 4.1. Comparison of Pore Structure Determined by MIP and N_2_ Adsorption

**Figure 12:**
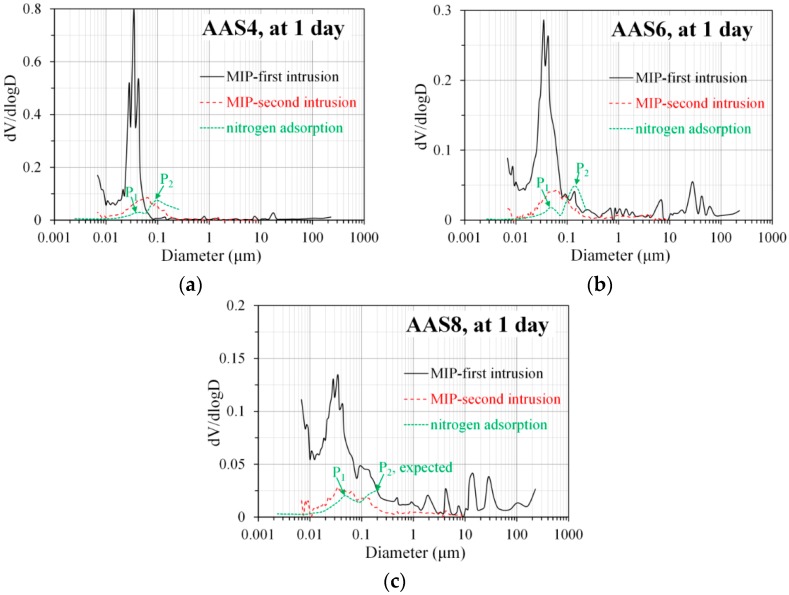
Comparison of the differential curves derived from MIP and N_2_ adsorption for AAS4 (**a**), AAS6 (**b**), and AAS8 (**c**) at 1 day. In the graphs, P_1_ and P_2_ are the first and second peak respectively, AAS indicates alkali-activated slag, and the number following AAS refers to the weight percentage of Na_2_O with respect to slag. For all samples, the water to slag ratio was 0.4 and the curing temperature was 20 °C.

For a clear comparison and discussion, Figure 12 shows the differential curves derived from MIP and N_2_ adsorption for AAS4, AAS6, and AAS8 at 1 day as an example. The differential curve derived from MIP (either the first intrusion or the second intrusion) shows only one peak in a pore size between 0.01 and 0.1 μm, corresponding to gel pores. However, the differential curve derived from N_2_ adsorption generally shows two peaks. At pore sizes smaller than 0.01 μm, the differential curve derived from MIP potentially implies a peak. However, the differential curve derived from N_2_ adsorption, at such pore sizes (<0.01 μm), is almost horizontal, showing no appearance of a peak. The indication of existence of a peak at pore diameter smaller than 0.01 μm by MIP, can result from the damage caused by the high pressure used during MIP measurements [34]. Actually, even at relatively low pressures of 10–20 MPa, damage was found in the interior of the sample [38].

Particularly, the differential curve of AAS4 shows two peaks in the pore sizes between 0.01 and 0.1 μm, corresponding to gel pores. This phenomenon was also observed by Ma et al. in a comparative study of the pore structure of alkali-activated fly ash using MIP and nitrogen adsorption [19]. Those authors attributed this difference to the different mediums (mercury compared to nitrogen) and different principles (liquid intrusion in comparison with gas adsorption) used in MIP and N_2_ adsorption measurements. It is noted that the damage caused by pressures may also contribute to the appearance of only one peak corresponding to the gel pores in MIP results.

The first peak in the differential curves of AAS6 and AAS8, derived from N_2_ adsorption, is in a pore size between 0.01 and 0.1 μm and corresponds to gel pores, which is in line with the peak observed from the differential curve derived from MIP. The second peak derived from N_2_ adsorption has a pore size larger than 0.1 μm and corresponds to capillary pores, which is, however, not identified from MIP results. The absence of the second peak in the differential curve derived from MIP may be caused by the “ink-bottle” effect during the MIP measurements. Due to the “ink-bottle” effect, MIP measures larger pores inside the sample only by intruding mercury through smaller bottlenecks. As a result, a significant proportion (>60%) of large pores with a pore size larger than 0.1 μm are counted as small pores with a pore size smaller than 0.1 μm [26]. The elimination of such a significant proportion of large pores (>0.1 μm) from the pore size distribution may lead to the disappearance of the second peak in the differential curve.

### 4.2. Comparison of Pore Structure Derived from MIP and Image Analysis

**Figure 13:**
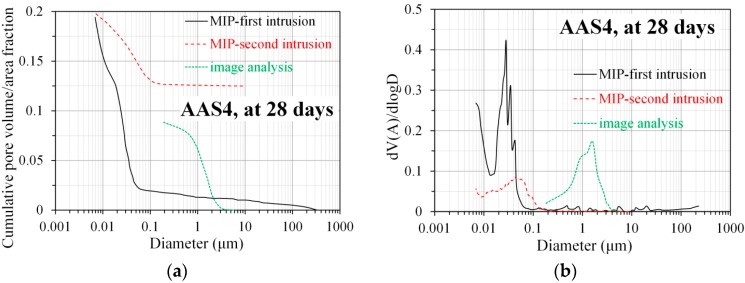
Comparison of the pore size distribution (**a**) and differential curve (**b**), derived from MIP and image analysis, for AAS4 at 28 days. In the graphs, AAS indicates alkali-activated slag, and the number following AAS refers to the weight percentage of Na_2_O with respect to slag. For all samples, the water to slag ratio was 0.4 and the curing temperature was 20 °C.

As an example, the pore size distribution and differential curve, derived from MIP and image analysis, for AAS4 at 28 days are shown in Figure 13. It is clear that the pore sizes obtained from image analysis are two orders of magnitude larger than those obtained from MIP. Compared to the total pore area fraction obtained from image analysis, MIP measures of total porosity were about two times larger. All those observations are in agreement with the findings in Portland cement paste [16,17]. Because of the “ink-bottle” effect, the volume of larger pores is counted as the volume of smaller pores, which leads to an underestimation of large pores and an overestimation of small pores [39]. For this reason, the pore size distribution obtained from MIP shift to small pore sizes. On the contrary, the pore size distribution obtained from image analysis represents the sizes of the pores actually visible with SEM. Because of the resolution limit of BSE images, the image analysis cannot detect the pores with diameter smaller than 0.1786 μm. As such, the total pore area fraction obtained from image analysis does not take all pores into account, and as a result, it is smaller than the total porosity obtained from MIP.

It is clear that the threshold pore diameter of gel pores obtained from the first MIP intrusion (0.028 μm) is smaller than that obtained from the second MIP intrusion (0.043 μm). Compared to the first MIP intrusion, the second MIP intrusion has little ink-bottle effect and provides a more truthful size distribution of pores [40]. In other words, the pore sizes obtained from the second MIP intrusion are little underestimated. Thus, the threshold pore diameter of gel pores obtained from the second MIP intrusion is larger. The pore diameter that corresponds to the peak derived from image analysis (1.6 μm) is about two orders of magnitude larger than the threshold pore diameters of gel pores obtained from the first MIP intrusion (0.028 μm) and the second MIP intrusion (0.043 μm). This result is expected, since the capillary pores have larger pore sizes than the gel pores.

### 4.3. A Brief Summary of MIP, N_2_ Adsorption, and Image Analysis in Characterizing the Pore Structure of Sodium Hydroxide Activated Slag

MIP is capable of detecting the total porosity of sodium hydroxide activated slag, but it is not able to provide the real pore size distribution due to the “ink-bottle” effect. Because of this intrinsic limitation, the pore size distribution derived from MIP normally underestimates the large pores while overestimates the small pores. As such, the pore size distribution derived from MIP should be interpreted carefully, and it is not feasible to combine the pore size distributions derived from MIP, N_2_ adsorption, and image analysis together, so as to obtain the pore size distribution in the whole range of pore sizes. Furthermore, the “ink-bottle” effect may even result in the absence of the second peak, corresponding to capillary pores, from the differential curve of MIP pore size distribution. In this situation, N_2_ adsorption is preferred since it can detect the second peak (see Figure 12), and additionally provides more information on nanopores. As seen in Figure 13, image analysis is able to eliminate the “ink-bottle” effect and reveals the real pore structure, especially in terms of capillary pores. However, the minimum pore size that image analysis can detect is limited to the resolution of the SEM images obtained.

### 4.4. Microstructure Formation of Sodium Hydroxide Activated Slag Paste

**Figure 14:**
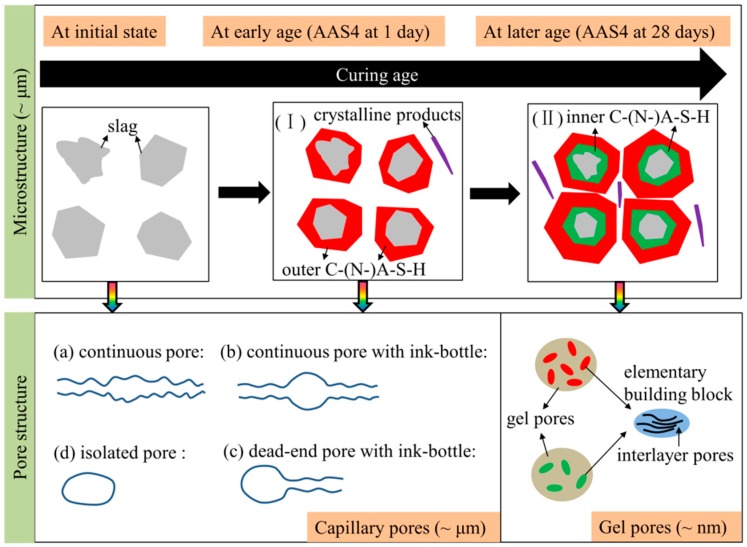
Schematic illustration of the microstructure formation and pore structure of sodium hydroxide activated slag samples.

The microstructure formation of sodium hydroxide activated slag is schematically illustrated in Figure 14. When slag is brought into contact with an alkaline activator, a set of reactions start and subsequently various solid reaction products are formed. According to the observations from SEM images (see Figure 5), two stages can be identified during the microstructure formation process of sodium hydroxide activated slag. In the first stage (I), the high initial rate of reaction does not allow time for diffusion of the dissolved ions and the reacting slag grains provide nucleation sites, as a result of which, the reaction products are built up in the zone immediately surrounding the slag grains. The reaction products, e.g., outer C–(N)–A–S–H, formed in this stage grows with continuous reaction of slag. In the second stage (II), the reaction products mainly develop in the place of the original slag particles, forming the inner layer of reaction products surrounding the slag grains, e.g., inner C–(N)–A–S–H. According to the grey level in the BSE image, it can be inferred that the outer C–(N–)A–S–H formed in the early stages of reaction is denser than the inner C–(N–)A–S–H formed at later stages. Along with those two stages, secondary reaction products, such as hydrotalcite phase, are formed in the empty pore space in the matrix, as reported in Ref. [23].

With continuous growth of primary reaction products around reacting slag grains and secondary reaction products in the empty pore space, the volume of capillary pores is gradually decreased and the sizes are refined with time. As seen in Figure 14, a capillary pore can be briefly classified into four types [17]: (a) continuous pore, (b) continuous pore with ink-bottle, (c) dead-end pore with ink-bottle, and (d) isolated pore. In MIP measurements, all pore space will be filled with mercury in the first intrusion. When the pressure is released, the mercury is sucked out of the pores except for the ink-bottle pores and isolated pores. It means that the ink-bottle pores and isolated pores do not contribute to the effective porosity, as derived from the second intrusion curve. With continuous reaction of slag, the ink-bottle pores may be transferred into continuous pores, which will be discussed in detail later.

Like C–S–H in in Portland cement-based materials, C–(N–)A–S–H in alkali-activated slag has a layered structure similar to that of disordered tobermorite-like phase [7,8]. Both C–S–H and C–(N–)A–S–H can be assumed to be made of elementary building blocks and gel pores. The elementary building block contains interlayer pores where water is chemically bonded to the C–S–H or C–(N–)A–S–H platelets, as seen in Figure 14. The elementary building block that can be used to build up the nanometer structure of C–S–H has been conceptualized by varieties of shapes and sizes, such as brick-shaped particles with size of 60 × 30 × 5 nm^3^ [41] and disk-shaped particles with a radius of 9.5 nm and a thickness of 0.113 nm [42]. Compared to C–S–H, C–(N–)A–S–H has a much more tightly packed atomic structure [43,44]. Therefore, the elementary building block of C–(N–)A–S–H should have smaller dimensions and thus a larger density than those of C–S–H. It is reported that the inner C–(N–)A–S–H has a higher Na content and more chemical bound water than the outer C–(N–)A–S–H [23]. This indicates that the elementary building block in the inner C–(N–)A–S–H is different from that in the outer C–(N)–A–S–H. This finding is different from the fact that the high-density C–S–H and low-density C–S–H are made of the same elementary building blocks in Portland cement-based materials [45]. Because the outer C–(N–)A–S–H has a larger density than the inner C–(N)–A–S–H, the elementary building block in outer C–(N–)A–S–H has a higher packing density than that in inner C–(N)–A–S–H. Therefore, the outer C–(N–)A–S–H has a smaller gel porosity than the inner C–(N)–A–S–H.

**Figure 15:**
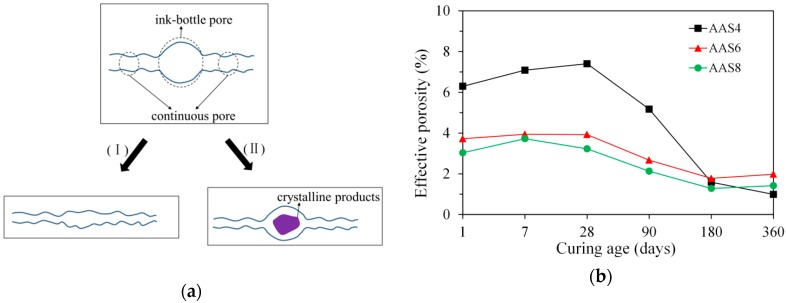
Schematic illustration of the transformation of ink-bottle pores into continuous pores (**a**), and effective porosity of samples for AAS4, AAS6, and AAS8 (**b**). In the graph (**b**), AAS indicates alkali-activated slag, and the number following AAS refers to the weight percentage of Na_2_O with respect to slag. For all samples, the water to slag ratio was 0.4 and the curing temperature was 20 °C.

As illustrated in Figure 15a, the ink-bottle pores may be transferred into continuous pores with continuous reaction of slag, leading to an increase of effective porosity. In the first case (I), the growth rate of reaction products on the inner wall of ink-bottle pore may be larger than that on the inner wall of continuous pores. In this situation, the size of ink-bottle pore may decrease to a size that is comparable to the size of continuous pores and therefore the ink-bottle pore does not exist anymore. In the second case (II), some secondary reaction products, such as hydrotalcite phase, may form in the ink-bottle pore (see Figure 14). The formation of secondary reaction products may segment the ink-bottle pores into small pores with sizes comparable to the sizes of continuous pores. As a result, the ink-bottle pore may disappear. The transformation of ink-bottle pores into continuous pores can be supported by the decrease of ink-bottle porosity (see Figure 9a) and increase of effective porosity at early age (see Figure 15b). Due to the increase of effective porosity, pore connectivity increases with time at the early age up to 28 days (see Figure 9b).

### 4.5. Pore Space Filling Capacity

**Figure 16:**
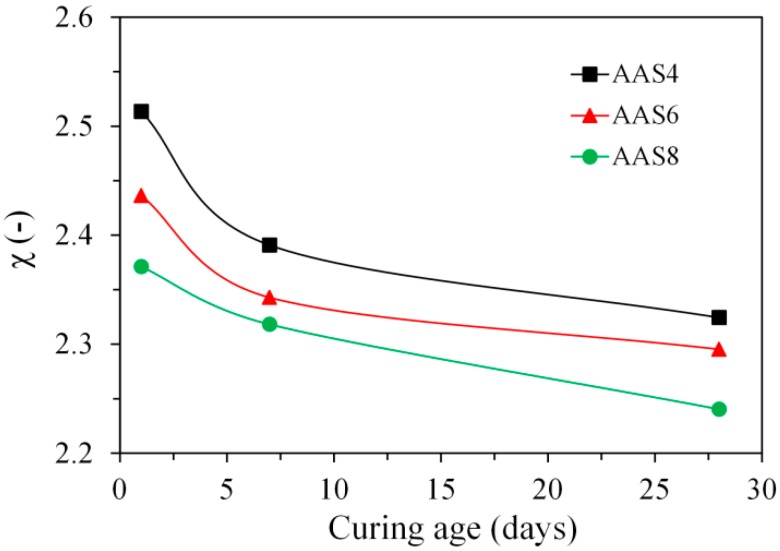
The volume ratio (*χ*, *V_products_*/*V_slag-reacted_*) of the reaction products relative to the reacted slag for the samples of AAS4, AAS6, and AAS8 at 1, 7, and 28 days. In the graph, AAS indicates alkali-activated slag, and the number following AAS refers to the weight percentage of Na_2_O with respect to slag. For all samples, the water to slag ratio was 0.4 and the curing temperature was 20 °C.

In the calculation of the volume ratio (*χ*, *V_products_*/*V_slag-reacted_*) according to Equation (10), the total porosity determined by MIP and the degree of reaction of slag determined by image analysis were used. Figure 16 plots the volume ratio (*χ*) as a function of curing age for the samples of AAS4, AAS6, and AAS8. It can be seen that *χ* falls between 2.24 and 2.51, which is a little bit larger than the value reported for Portland cement from 2.06 to 2.2 [46]. This indicates that the reaction products in sodium hydroxide activated slag has a higher pore space filling capacity than that in the Portland cement, which therefore leads to a denser microstructure in sodium hydroxide activated slag than in Portland cement at the same initial porosity.

The volume ratio (*χ*) decreases with curing age for all the sodium hydroxide activated samples. This result is expected, since the growth of reaction products at later age is confined by the reaction products formed at early age, for example, inner C–(N–)A–S–H confined by outer C–(N–)A–S–H as seen in Figure 5 and Figure 14. As the Na_2_O content increases, the volume ratio decreases. The increase of Na_2_O content leads to a higher alkalinity of sodium hydroxide activator, which has two effects that result in the decrease of *χ*. On one hand, the higher alkalinity of sodium hydroxide activator enhances the initial rate of reaction and thus accelerates the formation of outer C–(N–)A–S–H. The rapid formation of outer C–(N–)A–S–H makes it hard for further reaction products to form in the empty coarse pores, but instead in the place of original dissolved slag. This can be seen from the comparison between the microstructures of AAS8 (Figure 5e) and AAS6 (Figure 5c) at 1 day. On the other hand, the higher alkalinity of sodium hydroxide activator results in a higher polymerization degree of C–(N–)A–S–H [47]. A higher polymerization degree of C–(N–)A–S–H reduces the amount of chemically bound water in C–(N–)A–S–H [23] and therefore leads to a higher density of C–(N)–A–S–H. For this reason, the pore space filling capacity is reduced, which is reflected by the decrease of the volume ratio (*χ*).

## 5. Conclusions

In this study, the pore structure of sodium hydroxide activated slag paste cured for different time up to 360 days was investigated through MIP, N_2_ adsorption, and SEM-image analysis. Based on the experimental results and discussions, the following conclusions can be drawn:

1. MIP: The total porosity, from an initial value of 54.2%, drops about 70% within the first day and then decreases slowly with time to 7–10% at 360 days. The ink-bottle porosity decreases continuously with time, while the pore connectivity increases with time at early stages up to 28 days and then decreases until 360 days. For all the samples from 1 to 360 days, at most one peak that corresponds to gel pores was identified in the differential curves. As the Na_2_O content and curing age increase, the identified peak shifts to a smaller pore diameter.
2. N_2_ adsorption: The porosity of small pores (<0.25 μm) increases with time at early stage, for example, up to 28 days for AAS4, and then decreases till 360 days. An increase of Na_2_O content leads to a lower porosity of small pores. In general, the differential curves show two peaks, and the trend that pore diameters of those two peaks vary with curing age depends on the content of Na_2_O.
3. SEM-image analysis: The degree of reaction of slag is higher for samples with longer curing time and higher content of Na_2_O. About 50% of slag was reacted within the first day. The peak identified in the differential curves is found at about 1.6 μm, and it shows little change with increasing curing age and Na_2_O content.
4. MIP vs. N_2_ adsorption: The comparison between differential curves at pore sizes smaller than 0.01 μm reveals damage resulting from high pressure during MIP measurement. The “ink-bottle” effect may lead to the absence of the second peak that corresponds to the capillary pores in the MIP results.
5. Microstructure formation: The increase of Na_2_O content and curing age led to a reduced porosity and a refined microstructure. Conceptual models are proposed to describe the microstructure formation process, during which two layers of reaction products, e.g., outer C–(N–)A–S–H and inner C–(N)–A–S–H, grow successively around the reacting slag grains, while the secondary reaction products, such as the hydrotalcite phase, are formed in the empty coarse pore space.
6. Pore space filling capacity: Sodium hydroxide activated slag has a higher pore space filling capacity than Portland cement. Along with the increases of Na_2_O content and curing age, the pore space filling capacity of sodium hydroxide activated slag decreases.

## Figures and Tables

**Table 1:** Chemical composition of blast furnace slag.

Oxide	SiO_2_	CaO	Al_2_O_3_	MgO	Fe_2_O_3_	SO_3_	K_2_O	TiO_2_	L.I. *
Weight (%)	32.91	40.96	11.85	9.23	0.46	1.61	0.33	1.00	1.15

* L.I. refers to loss on ignition.

**Table 2:** Mix composition of the alkali-activated slag pastes.

Mix	Slag (g)	Na_2_O (g)	Water (g)
AAS4	100	4	40
AAS6	100	6	40
AAS8	100	8	40

**Table 3:** Porosity (%) of small pores (<0.25 μm) derived from nitrogen adsorption.

Samples	1 Day	7 Days	28 Days	180 Days	360 Days
AAS4	5.98	6.89	7.19	6.50	4.87
AAS6	3.85	3.46	2.45	1.57	1.81
AAS8	2.82	3.22	2.50	1.07	1.19

## Supplementary Materials

The following are available online at http://www.mdpi.com/1996-1944/11/6/1035/s1, Figure S1: Pore size distribution and differential curves, derived from MIP, for AAS4, AAS6 and AAS8 at 7, 90 and 180 days. In the graphs, P_1_ and P_2_ are the first and second peak respectively (here P_2_ is not identified), AAS indicates alkali-activated slag, the number following AAS refers to the weight percentage of Na_2_O with respect to slag. For all samples, the water to slag ratio was 0.4 and the curing temperature was 20 °C, Figure S2: Pore size distribution and differential curves, derived from nitrogen adsorption, for AAS4, AAS6 and AAS8 at 7 and 180 days. In the graphs, P_1_ and P_2_ are the first and second peak respectively, AAS indicates alkali-activated slag, the number following AAS refers to the weight percentage of Na_2_O with respect to slag. For all samples, the water to slag ratio was 0.4 and the curing temperature was 20 °C.
