# Supplementary material for: Pore Structure Characterization of Sodium Hydroxide Activated Slag Using Mercury Intrusion Porosimetry, Nitrogen Adsorption, and Image Analysis
Source: Materials (Basel). 2018 Jun 19;11(6):1035. doi: 10.3390/ma11061035 (PMC6025311; doi:10.3390/ma11061035)
Supplement: Supplementary file 1 [file materials-11-01035-s001.pdf]

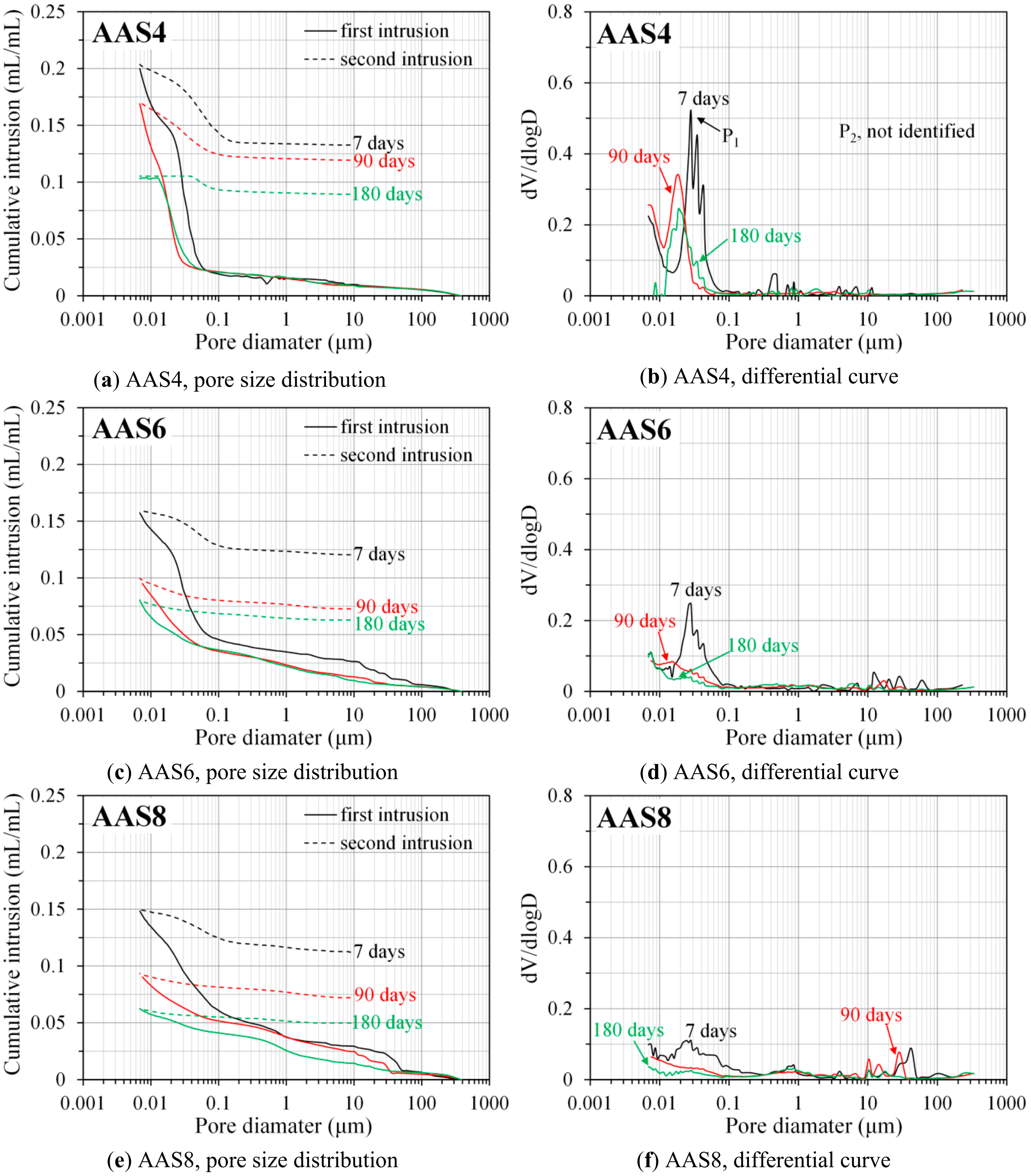

(**a**) AAS4, pore size distribution (**b**) AAS4, differential curve

(**c**) AAS6, pore size distribution (**d**) AAS6, differential curve

(**e**) AAS8, pore size distribution (**f**) AAS8, differential curve

Figure S1. Pore size distribution and differential curves, derived from MIP, for AAS4 (a, b), AAS6 (c, d) and AAS8 (e, f) at 7, 90 and 180 days. In the graphs, P_1 and P_2 are the first and second peak respectively (here P2 is not identified), AAS indicates alkali-activated slag, the number following AAS refers to the weight percentage of Na_2O with respect to slag. For all samples, the water to slag ratio was 0.4 and the curing temperature was 20 °C.

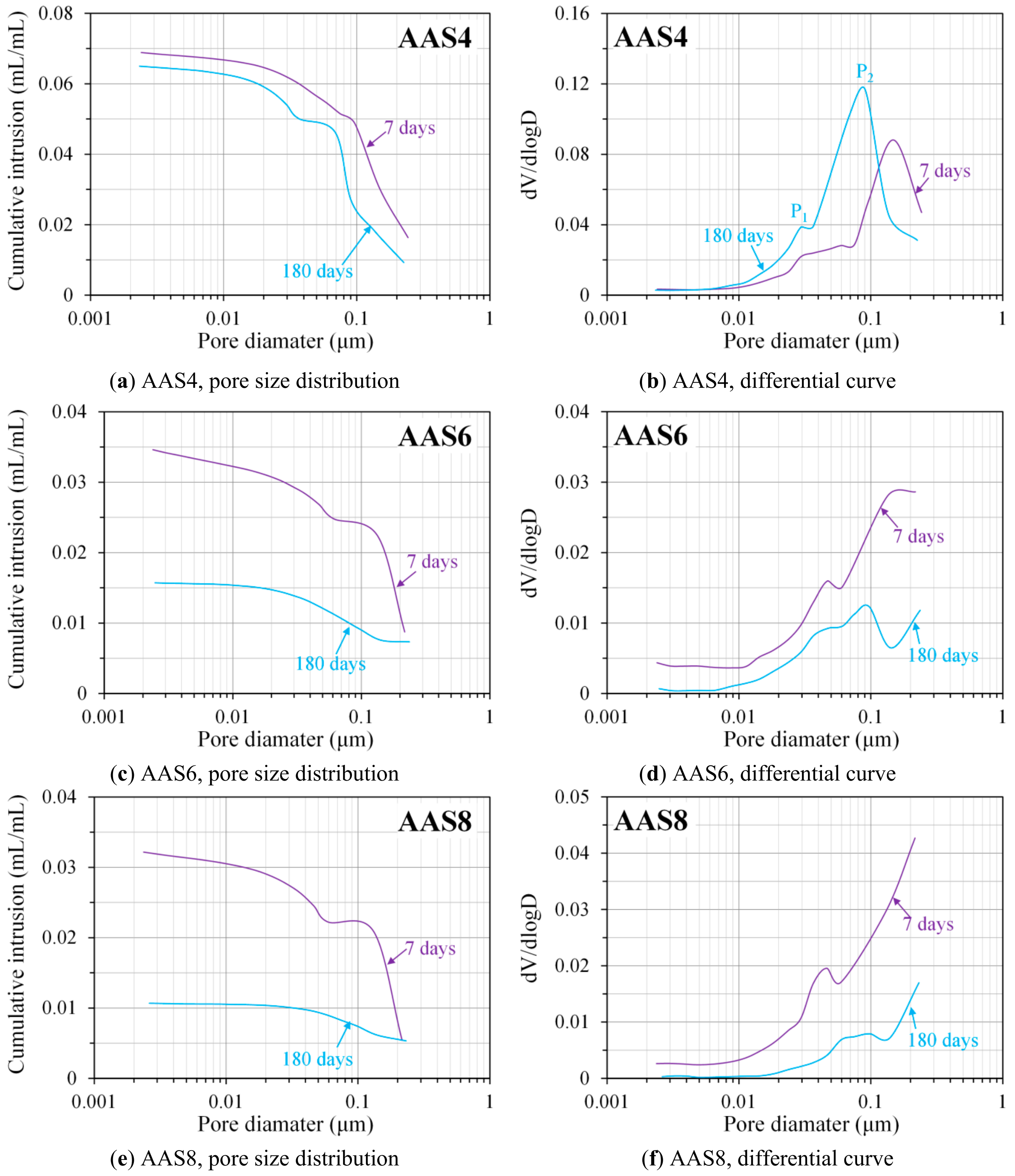

Figure S2. Pore size distribution and differential curves, derived from nitrogen adsorption, for AAS4 (a, b), AAS6 (c, d) and AAS8 (e, f) at 7 and 180 days. In the graphs, P_1 and P_2 are the first and second peak respectively, AAS indicates alkali-activated slag, the number following AAS refers to the weight percentage of Na_2O with respect to slag. For all samples, the water to slag ratio was 0.4 and the curing temperature was 20 °C.
